# Effect of Pyrolysis Temperature on the Performance of Malt Biochar in Cement Mortars

**DOI:** 10.3390/ma18225105

**Published:** 2025-11-10

**Authors:** Roza Shainova, Nelli Muradyan, Avetik Arzumanyan, Marine Kalantaryan, Rafayel Sukiasyan, Mkrtich Yeranosyan, Yeghvard Melikyan, Avetis Simonyan, David Laroze, Elisabetta Zendri, Manuk Barseghyan

**Affiliations:** 1 Faculty of Construction, National University of Architecture and Construction of Armenia, 105 Teryan Street, Yerevan 0009, Armeniaavetik.arzumanyan@nuaca.am (A.A.); manuk.barseghyan@nuaca.am (M.B.); 2 Innovation Center for Nanoscience and Technologies, A.B. Nalbandyan Institute of Chemical Physics NAS RA, 5/2 P. Sevak Str., Yerevan 0014, Armenia; 3 Instituto de Alta Investigación, Universidad de Tarapacá, Casilla 7D, Arica 1000000, Chile; 4 Department of Environmental Sciences, Informatics and Statistics, Ca’ Foscari University Venice, Via Torino 155, 30125 Venice, Italy

**Keywords:** malt, pyrolysis, biochar, cementitious mortar, compressive strength

## Abstract

**Highlights:**

**What are the main findings?**

- Malt biochar at 500 °C showed improved graphitic structure and fewer defects.
- Milled BC500 increased compressive strength by up to 20.6%.
- BC500 reduced water absorption, leading to denser mortars.
- Superplasticizer + BC500 gave the strongest performance (62 MPa).

**What are the implications of the main findings?**

- Malt waste valorization supports the circular economy in construction.
- Biochar enhances strength and durability of cement mortars.
- Use of BC lowers CO_2_ impact compared to conventional binders.
- Promotes sustainable, high-performance green building materials.

**Abstract:**

This study examines the influence of pyrolysis temperature on the physicochemical characteristics of malt-derived biochar (BC) and its effect on the performance of cement mortars. Malt biomass, a by-product of the brewing industry, was subjected to pyrolysis at 300 °C and 500 °C, followed by high-energy ball milling to produce nanoscale biochar. Characterization using FTIR, Raman spectroscopy, XRD, BET, SEM, and XRF revealed that BC500 possessed higher graphitic ordering, larger specific surface area (110 m_2_/g), and smaller pore size compared to BC300, which exhibited greater hydrophobicity. Incorporation of BC500 into cement mortars at 0.25–1.0 wt.%, with and without superplasticizer, resulted in up to a 20.6% increase in compressive strength and a significant reduction in water absorption. These enhancements are attributed to the internal curing effect of biochar, its refined pore structure, and improved interfacial bonding with hydration products. The findings demonstrate that optimized malt biochar serves as a sustainable additive that improves the mechanical performance and durability of cementitious materials while advancing circular economy principles through the valorization of industrial malt waste and the mitigation of the environmental impact of cement production.

## 1. Introduction

Cement plays a fundamental role in modern infrastructure, functioning as the key binding component in cement paste, mortar, and concrete. However, its production ranks among the most energy-intensive industrial activities, requiring significant thermal input and resulting in considerable greenhouse gas emissions [1,2]. A substantial portion of this environmental impact stems from the calcination of limestone during clinker production, which releases approximately 0.87 tons of CO_2_ for every ton of cement produced [3,4]. The cement industry is estimated to be responsible for nearly 8–9% of total global anthropogenic CO_2_ emissions, positioning it as a major contributor to climate change and one of the most environmentally impactful sectors in construction [5]. Global cement production reached approximately 4.2 billion tons in 2017 and is projected to increase to 4.5 billion tons by 2050, reflecting the continuing expansion of the global construction sector and intensifying the need for sustainable material alternatives [6]. This growth trajectory reflects the accelerating pace of global urbanization and infrastructure development, reinforcing the urgency of identifying and adopting sustainable alternatives. In light of these environmental and performance considerations, researchers have increasingly focused on understanding and improving the fundamental behavior of cementitious systems as a pathway to sustainability. In this context, recent studies have characterized the physical and chemical behavior of cementitious systems, emphasizing hydration, microstructure, and phase development as key determinants of mechanical strength and durability [7,8,9,10,11].

The current global policy aimed at conserving resources is based on developing both new and existing technologies, such as recycling and repurposing industrial waste in various ways [12,13]. Incorporating industrial by-products into binders and concrete to lower the percentage of Portland cement is crucial for preserving and enhancing the mechanical properties and lifespan of the final products [14]. The desired objectives can be accomplished by incorporating biologically based components into concrete, which, as established by well-known scientific studies, positively influence its strength properties [15]. In recent years, numerous investigations have demonstrated that supplementary materials such as fly ash, silica fume, and slag contribute to refined pore structures and denser hydration products, thereby enhancing the mechanical performance and durability of cementitious composites [16,17,18,19]. These studies emphasize that the microstructural evolution and phase assemblage of cement composites are strongly influenced by the chemical composition, concentration, and surface characteristics of the additives, as well as by factors such as the water–cement ratio and mixing methodology [20,21,22].

Another approach is to use BC, a carbon-rich material produced through biomass pyrolysis, in cement-based materials. According to the International Biochar Initiative, BC is a solid that is created when biomass is thermos-chemically converted into an oxygen-limited environment [23]. In 2016, the European Union produced approximately 50 million tons of wood waste, of which 49% was recycled, 2% was burned, 1% was disposed of in landfills, and 48% was burned with energy recovery. Compared to conventional burning, pyrolysis and gasification have far smaller environmental impacts. Significant energy recovery is made possible by their utilization, which can also enhance waste management and reduce the hazardous emissions associated with the removal of wood waste [24,25]. Accordingly, biochar production not only facilitates the valorization of biomass waste but also supports more energy-efficient and environmentally benign waste management practices. Recent reviews have affirmed the potential of biochar in the construction sector, particularly in its integration into cementitious matrices, which supports carbon sequestration and enhances the performance of composites [26,27,28].

The integration of BC has become a pioneering strategy in the concrete industry. As an eco-friendly additive, BC enhances the mechanical strength and durability of concrete while also facilitating carbon sequestration, thereby reducing the environmental impact of conventional cement production. By integrating BC into concrete mixes, researchers aim to develop eco-friendly construction materials with potential benefits in thermal insulation, water retention, and resistance to cracking [29,30,31]. These findings are further supported by recent experimental work showing improved hydration kinetics, refined pore distribution, and enhanced resistance to thermal transfer in biochar-modified mortars [32,33,34]. Because BC is made from different raw materials and produced in various facilities with distinct properties, there is no ideal mix design for its use. The morphology, carbon purity, volatile emissions, and pore size distribution of BC are greatly affected by the biomass type and production parameters, such as oxygen availability below the stoichiometric limit (pyrolysis or gasification), peak temperature, heating rate, and pressure [22,35,36]. Research has demonstrated that variations in pyrolysis conditions result in differing levels of aromaticity and surface reactivity, which in turn have a direct impact on cement hydration and setting behavior [26,37,38].

BC particles can undergo a variety of pretreatments before being added to cementitious mixtures, including presoaking, grinding, and screening. Each of these processes affects the interaction of BC with the cementitious matrix, producing generally promising outcomes [39]. These modifications impact the hydration mechanisms, influence setting times, and affect the durability of the composite material, ultimately enhancing its compatibility with the cement matrix and contributing to improved long-term structural performance. Microstructural analyses employing SEM, XRD, and XCT have revealed that well-dispersed, fine biochar particles facilitate the formation of C–S–H and mitigate microcracking by serving as nucleation sites [33].

The application of biochar as an admixture in concrete represents a growing innovation within the construction industry [35]. The article [36] reported that accelerated carbonation facilitates the formation of hydration products that subsequently convert into stable carbonates. The incorporation of biochar enhances this process by improving the pore structure of the concrete matrix, thereby promoting the diffusion of CO_2_ and accelerating the carbonation reaction. The effectiveness of biochar for use in concrete is largely influenced by both the pyrolysis conditions and the type of biomass feedstock employed [40]. An increase in pyrolysis temperature typically results in a higher carbon content while simultaneously reducing the overall yield of biochar [41]. Key characteristics—such as water retention capacity, pore size distribution, surface area, and cation exchange capacity—are determined by the nature of the biomass used as feedstock. Derived from plant biomass composed of hemicellulose, cellulose, and lignin, biochar’s properties are shaped by the distinct pyrolysis rates, mechanisms, and pathways of these components, leading to diverse porous structures and functional group formations. The adsorption capacity of biochar is based on various factors such as particle size, specific surface area, and the presence of functional groups like hydroxyl, carbonyl, and carboxyl, which are crucial for interactions within cementitious matrices [42,43]. Biochar is considered a suitable material for construction applications due to its high chemical stability. In addition, its inherent porosity contributes to low thermal conductivity, enhancing its insulating properties [44]. Nevertheless, the release of acidic functional groups and the formation of basic oxides during the pyrolysis process can raise the alkalinity of cementitious systems, potentially compromising long-term durability [45]. Moreover, utilizing biomass with a high mineral content can lead to reduced porosity, as excessive ash content may obstruct micropores [46]. Several recent investigations have quantified these relationships, linking specific surface area and pore morphology to strength retention and hydration behavior [11,22,47,48].

By optimizing these desirable characteristics and increasing the biochar content within the concrete matrix, biochar presents a promising route toward the development of high-strength, carbon-neutral concrete [48]. Previous research has utilized sophisticated characterization techniques, such as isothermal calorimetry, mercury intrusion porosimetry, and nanoindentation, to examine the interactions between biochar and cement. These methodologies have yielded significant insights into hydration kinetics, microstructural refinement, and the impact of biochar properties on mechanical performance [33,45,49].

One promising strategy for enhancing these effects is the production of nano-biochar through ball milling. Ball milling is an effective mechanical method for reducing biochar particle size and enhancing its surface characteristics, such as specific surface area and porosity [50]. Compared to untreated biochar, nano-biochar produced via ball milling exhibits significantly higher surface area and pore volume, which improves its reactivity and interaction within cementitious systems [51]. Current research is increasingly investigating physical activation techniques to modify the surface chemistry of biochar and enhance its compatibility with cement matrices [26].

Despite growing interest in the use of biochar in cementitious systems, most studies have focused on biochar derived from conventional feedstocks such as wood, straw, or nutshells. In contrast, barley malt residue, a by-product of the brewing industry, remains an underutilized and largely unexplored biomass source in the context of construction materials.

Due to its distinct organic composition, barley malt can yield biochar with specific surface characteristics and functional groups that may influence its interaction with cementitious matrices. However, to date, research on the use of milled barley malt-derived biochar in cement mortars is limited, and the influence of pyrolysis temperature on its properties and performance has not yet been comprehensively addressed. Moreover, while the interaction between biochar and superplasticizers has been discussed in the literature [52], there is limited research exploring the combined effect of food-industry-derived, milled biochar and superplasticizers on mortar hydration and strength development.

To address this gap, the current study sought to produce biochar from barley malt at two distinct pyrolysis temperatures (300 °C and 500 °C), followed by milling. The study further aimed to assess the impact of the resulting materials on the mechanical properties and durability of cement mortars, both with and without the incorporation of a superplasticizer.

This research contributes to the development of high-performance and sustainable cementitious composites and supports circular economy strategies through the valorization of brewing industry residues.

## 2. Materials and Methods

This study focuses on:

✓ BC preparation starting from malt and characterization of the final products;
✓ addition of milled BC in a cementitious material, and evaluation of the mechanical performance of BC-concrete.

### 2.1. BC Preparation Starting from Malt and Characterization of the Final Products

**Figure 1:**
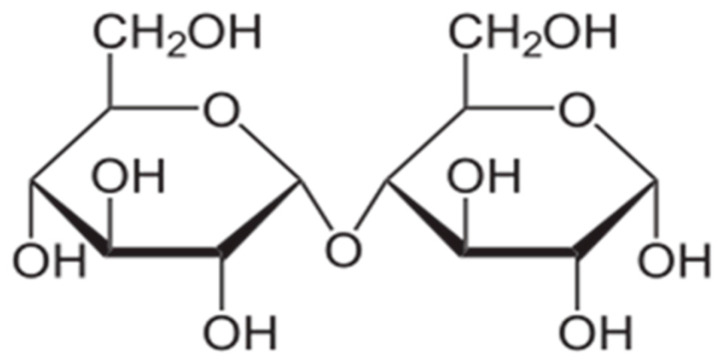
Chemical structure of maltose.

In this study, malt biomass was selected as the feedstock for biochar (BC) production due to its rich organic content and widespread availability as a brewing industry by-product. Before pyrolysis, the malt was first air-dried at room temperature to remove excess moisture, then oven-dried at 105 °C for 24 h to ensure complete dehydration. The dried biomass was subsequently ground to a fine powder using a laboratory mill and sieved to obtain particles smaller than 250 µm. This uniform particle size was essential to ensure consistent thermal decomposition and improve the efficiency of the pyrolysis process. The chemical structure of maltose, one of the primary sugar components in malt, is illustrated in Figure 1 [53].

The elemental and oxide composition of biomass used in this work is: P—0.9%, S—0.59%, Mn—0.005%, Cu—0.005%, Zn—0.01%, CaO—0.09%, K_2_O—0.4%, SiO_2_—0.0002%, Al_2_O_3_—2.67%, and Fe_2_O_3_—1.887%.

**Figure 2:**
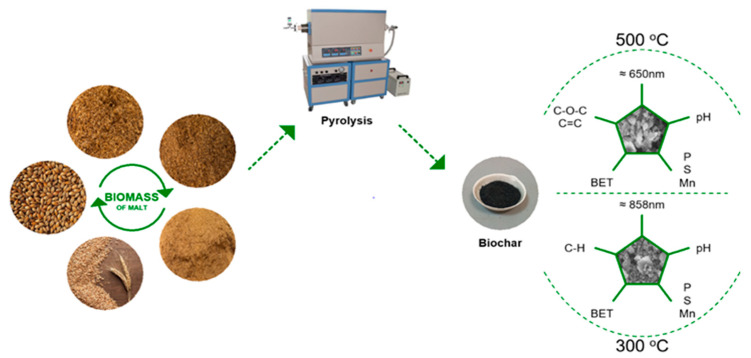
Pyrolysis of malt biomass at temperatures of 300 and 500 °C.

Figure 2 illustrates the pyrolysis-based production of BC from malt biomass. The process was carried out at two distinct temperatures, 300 °C and 500 °C, and the physicochemical properties of the resulting BC were analyzed.

BC was produced from malt rinds through pyrolysis at 300 °C and 500 °C in a horizontal tubular reactor under an argon gas flow rate of 500 sccm. The process was carried out at a heating rate of 10 °C/min with a holding time of 3 h, yielding BC with distinct chemical and physical properties depending on the temperature.

After pyrolysis, the resulting malt biochar (BC), which exhibited average particle sizes of approximately 858 nm at 300 °C and 650 nm at 500 °C (as shown in Figure 2), was further processed using a PM 100 laboratory planetary ball mill (RETSCH GmbH, Haan, Germany, maximum speed: 650 rpm) to achieve nanoscale refinement. For each milling batch, 6 g of biochar was placed into the milling container together with 14 stainless steel balls. The milling process lasted 70 min in total, comprising 30 min of clockwise rotation, a 10-min pause, and 30 min of counterclockwise rotation, operated at 500 rpm. This high-energy mechanical treatment is known to reduce particle sizes down to the nanoscale range (as low as approximately 100 nm), thus enabling the production of nanostructured biochar (nano biochar) that can potentially achieve improved dispersion and interaction within cementitious composites.

### 2.2. Sample Preparation of Cementitious Mortars

Portland cement M500 (52.5 N class, ARARAT CEMENT CORPORATION LLC, Ararat, Armenia) as a binder and river sand as a fine filler were used to prepare the cementitious samples. Melflux (BASF, Ludwigshafen, Germany), superplasticizer was utilized to reduce the water content of the mixtures, maintain the necessary plasticity, and enhance the dispersion of BC. Table 1 presents the mechanical and physical characteristics of Portland cement and its chemical composition [54,55,56] as available from the commercial datasheet.

River sand was utilized as the fine aggregate in the cement mortar production process, and Table 2 presents the average data of its physical properties, as available from the commercial datasheet [57].

A plasticizer (Melflux CC, BASF) was utilized to reduce the water content of the mixtures, maintaining the necessary plasticity. Ball-milled biochar (BC) obtained through pyrolysis at 300 °C were incorporated into the mortar mixture at concentrations of 0.25%, 0.50%, 1.00%, and 1.25% by cement mass, starting from the previous scientific results reported in the literature [58].

Similarly, ball-milled BC produced at 500 °C was incorporated at concentrations of 0.25, 0.50, and 1.00% by cement mass, and composites with a concentration of 1.25% were not developed, since a decrease in compressive strength was already observed at a 1% concentration of biochar obtained at 500 °C. After 28 days of curing, the densities and water absorption of the test samples were measured, while compressive strengths were evaluated at both 7 and 28 days of curing. A total of 15 compositions (N1–N15) were developed, and for each composition, 6 samples were prepared.

In the processed mortars, W/C = 0.47 was determined to be the optimal water-cement ratio; plasticizer, utilized for BC dispersion, was applied at 0.05% by cement mass. The mixture was prepared in the following sequence: the cement and sand were combined in a mortar mixer (Mortar mixer, Matest, Treviolo, Italy) at 140 rpm for 2 min, after which the dispersion of water with BC was introduced within 30–40 s. This dispersion was obtained through agitation with a magnetic stirrer for 2 min (Magnetic stirrer MM-5, Matest, Treviolo, Italy), and the mixing process continued for an additional 3 min. The remaining mixture was added, and after 1 min of stirring, the process was halted. The walls of the container were cleaned, and mixing resumed at a high speed of 285 rpm for 1 min, followed by 1 min of low-speed mixing. The completed mixture was subsequently transferred into metal molds.

**Figure 3:**
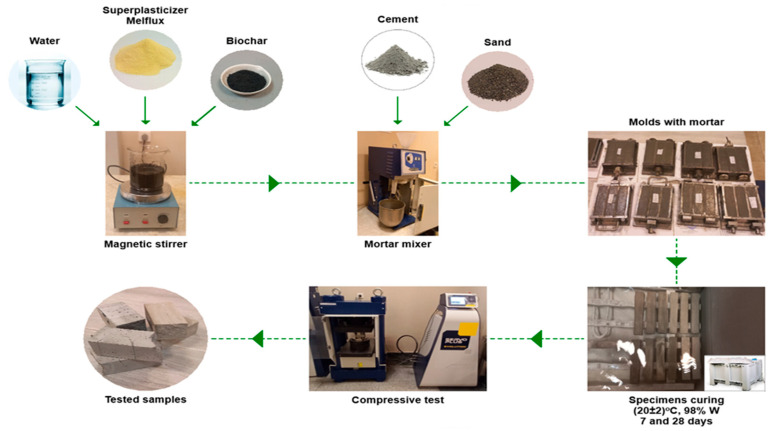
Preparation of mortar mixtures and test specimens.

As a result, six test specimens for each composition were prepared in prism shapes with dimensions of 40 × 40 × 160 mm. After 24 h, the specimens were removed from the molds and placed in a storage chamber set to a temperature of (20 ± 2) °C and (98 ± 2)% relative humidity for 28 days. The compressive strengths of the specimens were assessed at 7 and 28 days, along with their water absorption at 28 days, as listed in Section 2.3.2. When the plasticizer was incorporated into the mixture, the entire mixing procedure was replicated in the same manner, with the exception that the plasticizer was added to the water and bio coal dispersion and homogenized with a magnetic stirrer for 2 min. The processes involved in the production of mortars are illustrated in Figure 3: combining the raw components, preparing cement mortar, casting the test specimens, curing them under humid conditions, and subjecting them to compressive strength testing.

### 2.3. Methods

#### 2.3.1. Physical and Chemical Properties of BC

The BC was analyzed using several physical and spectroscopic methods. Specifically, XRD analysis (using a Rigaku MiniFlex instrument, Rigaku Corporation, Tokyo, Japan) provided crystallographic data. The chemical composition was investigated by ATR-FTIR spectroscopy (Spectrum Two device from PerkinElmer, Springfield, IL, USA). Bond types and graphitization of carbon black of the material were identified via AFM-combined Raman spectroscopy (LabRAM HR Evolution by HORIBA, Evanston, IL, USA). The particle size of the synthesized material was measured using DLS (Litesizer 500 by Anton Paar, Graz, Austria). The elemental composition of BC was analyzed using a Rigaku NEX DE X-ray fluorescence (XRF) system (Rigaku Corporation, Tokyo, Japan) combined with a JEOL JCM-7000 scanning electron microscope (SEM, JEOL JCM-7000, JEOL, Tokyo, Japan). BC pH and electrical conductivity were determined by preparing a homogeneous aqueous suspension according to a BC/deionized water weight ratio of 1:10, and after 30 min of stirring in a beaker with a magnetic stirrer. The pH and electric conductivity were measured by HACH LANGE HQ 14 d (Danaher Corporation, Düsseldorf, Germany).

After drying and degassing processes, the adsorption surface area measurements were conducted using a Micromeritics ASAP 2020 Plus instrument (Micromeritics Instrument Corporation, Singapore) with nitrogen as the adsorbate.

#### 2.3.2. Physical and Mechanical Characterization of Mortars

Water Absorption measurement—After 28 days, water absorption of cement mortars with BC was determined according to the GOST 12730.3-2020 [59] standard. The standard test method involves weighing a dry sample, submerging it in water for 24 h, and then weighing it again to calculate the percentage of water absorbed. The water temperature should be maintained at (20 ± 5) °C to ensure consistent and reliable results during the immersion process. To determine the water absorption, for the laboratory tests we used Italian “Matest” manufacturer equipment, such as measuring calipers with 0.01 mm accuracy and V073-01 balance with 0.1 g accuracy․

Compressive Strength Testing—The average compressive strength of six cubes from each batch was measured by a Concrete Compression Machine (Matest) 2000 kN automated, Servo-Plus Progress, following the standard EN 196-1 [55]. The specimens that were tested for compressive strength had dimensions of 40 × 40 × 160 mm, guaranteeing a uniform and consistent assessment procedure. The compressive tests were carried out at two time points, namely after curing for 7 and 28 days. Compressive tests were conducted utilizing an automatic pressure machine with a loading rate of 2.4 kN/s [55].

## 3. Results and Discussion

### 3.1. BCs Characterization

The BCs obtained from the pyrolytic process described in Section 2.1 have been characterized, and the results are shown below.

#### 3.1.1. ATR-FTIR Spectroscopy

**Figure 4:**
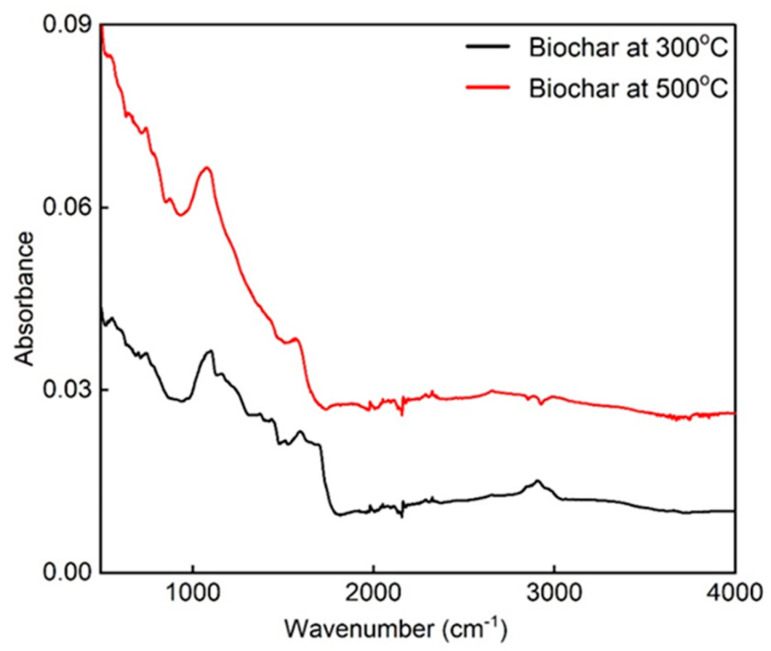
Fourier-transform infrared (FTIR) absorption spectra of the BC at 300 °C and 500 °C.

Figure 4 reports the FTIR-ATR spectra of the BCs obtained by pyrolysis at different temperatures. The ATR-FTIR spectra show that BC produced at 300 °C is predominantly characterized by functional groups typical of oxygenated hydrocarbons, which are associated with the carbohydrate structures of cellulose, hemicellulose, and lignin, to a greater extent than BC produced at 500 °C [60].

At 300 °C, the pyrolysis process produces non-polar aliphatic hydrocarbons or waxy residues on the BC. These residues make the BC hydrophobic despite the presence of oxygenated functional groups. These compounds often form as intermediate pyrolysis products, especially if the feedstock is rich in lipids or other hydrophobic organic components.

At 500 °C, the hydrophobic aliphatic residues decompose or volatilize, as reported in the spectrum in Figure 3. Simultaneously, new oxygenated functional groups (e.g., hydroxyl, carboxyl, or carbonyl groups) may form on the surface due to partial oxidation or chemical rearrangement. These groups enhance hydrophilicity by forming hydrogen bonds with water molecules [61]. The spectra also display the stretching of the stable aromatic BC backbone structure (C=C) and the asymmetric ether bond (C–O–C) at 1096 cm^−1^ and 1610.4 cm^−1^, respectively, for BC produced at 300 °C, and at 1074 cm^−1^ and 1578.7 cm^−1^ for BC produced at 500 °C [62,63].

These chemical modifications significantly influence the surface chemistry and wettability of the biochar, which in turn affect its interaction with water and cement hydration products. The hydrophobic nature of BC300 may reduce water availability during cement setting, whereas the increased hydrophilicity of BC500 can facilitate better water retention and promote internal curing effects. This difference is critical for understanding the distinct mechanical performance and durability enhancements observed in mortars containing biochar produced at these two temperatures. Furthermore, the presence of aromatic structures contributes to the thermal stability and chemical resistance of BC, supporting its role as a durable additive in cementitious systems.

#### 3.1.2. Raman Spectra

**Figure 5:**
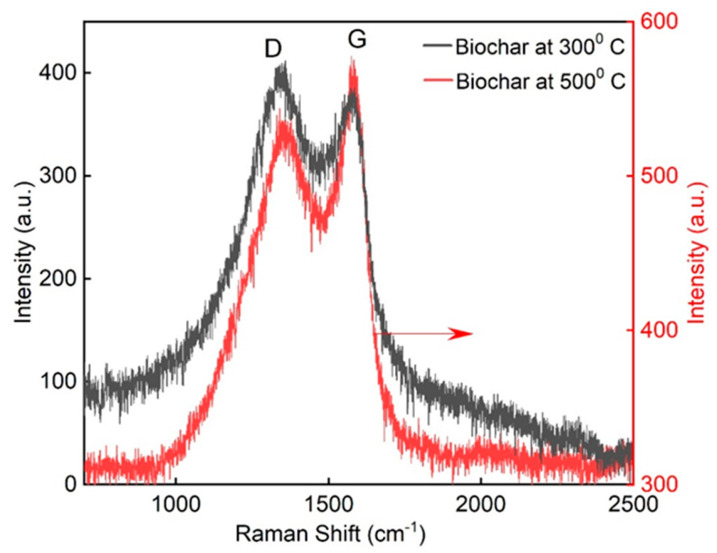
Raman spectra of BC at temperatures of 300 °C and 500 °C.

Figure 5 illustrates the Raman spectra of BC produced at 300 °C and 500 °C. Both BC samples exhibited the two characteristic peaks at 1352.7 cm^−1^ (D-bands) and 1579 cm^−1^ (G-bands). The D peak indicates the presence of defects and disordered structures within the carbon matrix, and the G band is associated with the vibrations of sp^2^-hybridized carbon atoms in graphitic carbon layers [64]. The ratio between D and G peaks (ID/IG) indicates the degree of graphitization of the BC. The sample pyrolyzed at 500 °C showed a relatively lower ID/IG ratio (0.85) compared to that at 300 °C (1.08), indicating a reduction in structural defects and enhanced graphitic ordering at higher temperature. This change in the ratio indicates that graphitization occurs at higher temperatures; additionally, the G band became more intense and narrower, consistent with the development of more ordered sp^2^ carbon structures [65,66].

Both spectra exhibited these findings, confirming that increasing the pyrolysis temperature leads to a greater degree of carbonization and structural ordering in biochar, which may improve its physicochemical properties for applications such as adsorption, catalysis, or as a reinforcing agent in composite materials.

Furthermore, the enhanced graphitic structure observed in BC500 is likely to improve its mechanical stability and chemical inertness when incorporated into cementitious matrices, thereby contributing to the observed increases in compressive strength and durability. The reduced defect density may also influence the surface interactions with hydration products, potentially promoting better particle–matrix bonding and limiting degradation under alkaline conditions. These structural characteristics support the suitability of high-temperature biochar as a functional additive for improving the microstructure and performance of cement-based materials.

#### 3.1.3. X-Ray Diffraction (XRD)

**Figure 6:**
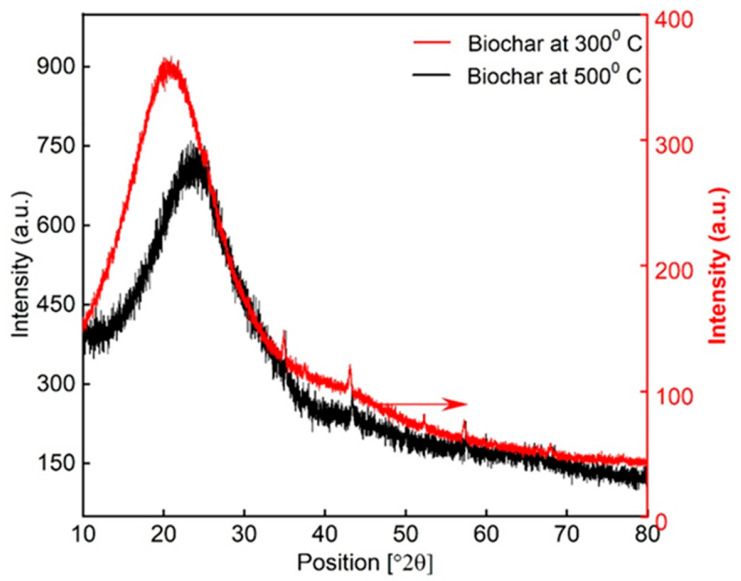
X-ray diffraction (XRD) spectra of BC at temperatures of 300 °C and 500 °C.

As shown in Figure 6, the pyrolysis temperature plays a crucial role in determining the structural and mineralogical characteristics of the resulting biochar. At 300 °C, the presence of distinct crystalline mineral peaks (33.5°, 43°, and 57°) suggests that the inorganic components in the malt biomass, such as potassium or calcium salts, remain relatively intact and crystallized. In contrast, the disappearance or significant reduction in these peaks at 500 °C implies that these mineral phases may undergo decomposition, transformation into amorphous forms, or integration into the carbon matrix [67,68,69].

The shift in the C (002) peak from 20.7° to 24.3° with increasing temperature further supports structural reorganization of the carbon matrix. This upshift is indicative of a decrease in the interlayer spacing between carbon sheets, suggesting enhanced graphitic ordering and development of turbostratic carbon structures. Moreover, the observed broadening and intensity reduction in the diffraction peak at ~22° at 500 °C points to a reduction in amorphous carbon content and a concurrent increase in aromatic condensation and carbon stacking [70,71].

Overall, the XRD analysis highlights that higher pyrolysis temperatures promote partial carbon ordering and structural transformation, leading to a more thermally stable and more electrically conductive biochar. These changes are significant for tailoring biochar properties for specific applications such as adsorption, catalysis, or use in cementitious composites.

#### 3.1.4. Particle Size Distribution

**Figure 7:**
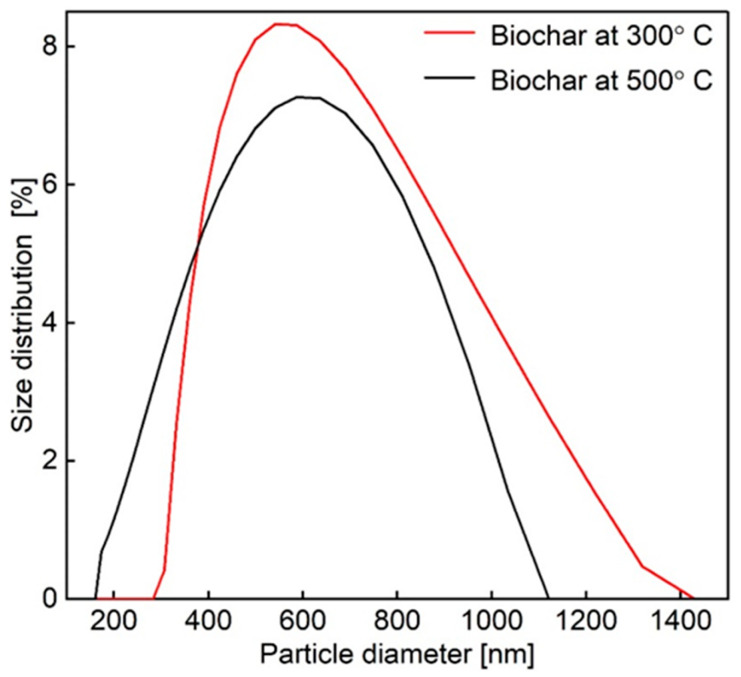
Particle size distribution of BC at temperatures of 300 °C and 500 °C.

The obtained average diameter distribution function for biochar particle sizes is presented in Figure 7․ As shown, the hydrodynamic diameter of biochar particles produced at 300 °C was approximately 491 nm, with a peak intensity at 535 nm (100%) and a standard deviation of 120 nm. In comparison, the sample produced at 500 °C exhibited a larger hydrodynamic diameter of 551 nm, with a peak intensity at 350 nm (100%) and a standard deviation of 50 nm.

These results suggest that pyrolysis temperature affects both the average particle size and its distribution. At 300 °C, the broader size distribution (higher standard deviation) may be due to incomplete carbonization, leading to weaker structures that fragment more easily during milling [72]. In contrast, 500 °C results in narrower distribution and more uniform particles, likely due to greater thermal degradation and aromatic condensation, which improve structural stability [63,73].

Although the average diameter at 500 °C is slightly larger, the peak intensity shifts to smaller sizes (350 nm), indicating more uniform particles with fewer larger aggregates. This pattern reflects the balance between particle shrinkage and aggregation at higher temperatures [74].

#### 3.1.5. Surface Area and Porosity

**Figure 8:**
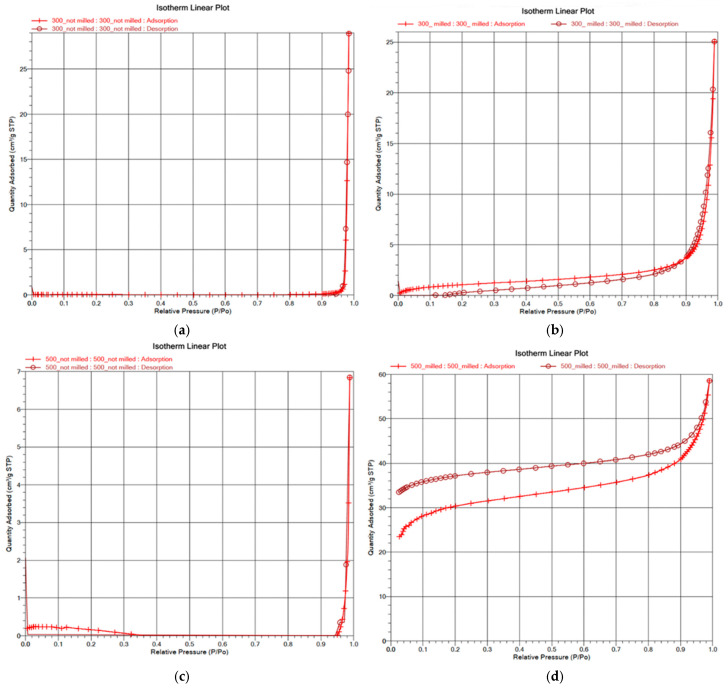
Isotherm Linear plots of unmilled BC300 °C (**a**) and 500 °C (**b**) pyrolyzed, Isothermal Linear plots of milled 300 °C (**c**) and 500 °C (**d**).

Figure 8 presents the relationship between relative pressure and the quantity adsorbed (cm^3^/g STP) for BC pyrolyzed at 300 and 500 °C—unmilled and milled. In Table 3, surface area and porosity data are presented. The unmilled 300 °C BC exhibits minimal adsorption within the 0–0.9 P/Po range, indicating negligible micro- (<2 nm) and mesoporosity (2–50 nm). The sharp rise in adsorption at 0.9 P/Po suggests some macroporosity (>50 nm), though the low surface area (0.1467 m^2^/g) confirms limited pore development and a rough surface. The absence of a hysteresis loop in the nearly identical adsorption/desorption isotherms further supports the lack of micro- and mesopores. While macroporosity is present, the low adsorption capacity highlights limited interaction and overall poor porosity.

Milled 300 °C BC exhibits an increased surface area (4.140 m^2^/g) due to milling, and the presence of smaller pores (~49.906 nm). The desorption curve is lower than the adsorption curve, suggesting the release of refractory organic compounds. This indicates that milling improves adsorption and opens previously blocked pores, allowing trapped organic compounds to evaporate during measurement [75].

The unmilled 500 °C BC exhibits a low surface area (1.081 m^2^/g) and limited adsorption capacity, indicating minimal micro- (<2 nm) and mesoporosity (2–50 nm) (Table 3, Figure 8c). The low adsorption curve reflects the restricted active surface area, while the sharp rise at 0.9 P/Po suggests the presence of macropores (>50 nm) and a rough surface. The close alignment of the adsorption and desorption curves indicates minimal retention of adsorbate, highlighting efficient desorption despite the macroporosity.

After the milling process of the sample, three main changes in the measurement results can be seen from the isotherm in Figure 8d:

✓ Significant increase in the adsorption in the low-pressure region;
✓ More smooth increase of adsorption in the high-pressure region;
✓ Mismatch between adsorption and desorption curves (hysteresis loop).

Milled 500 °C BC exhibits a surface area of 110.402 m^2^/g and small pores (2.349 nm). The h adsorption curve reflects strong adsorption potential, while the desorption curve surpassing the adsorption curve indicates that some adsorbed molecules remain trapped in micropores. This suggests that milling at a higher pyrolysis temperature creates smaller, well-connected micropores, improving adsorption efficiency and making it highly effective in mortars. The large surface area in BC provides more active sites for interaction with the hydration products of cement, such as calcium silicate hydrates (C-S-H). These interactions can enhance the bond between the BC particles and the cement matrix, contributing to improved mechanical properties, including hardness.

#### 3.1.6. Scanning Electron Microscope (SEM)

**Figure 9:**
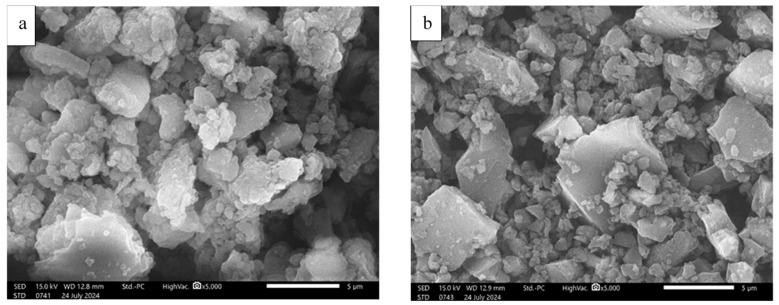
Scanning electron microscope images of malt BCs at 300 °C (**a**) and 500 °C (**b**).

Figure 9a,b present scanning electron microscope images of BC produced and milled at temperatures of 300 °C and 500 °C, respectively. The SEM images show that BC pyrolyzed at 300 °C undergoes a less complete carbonization process than that produced at 500 °C.

As shown in the SEM images, the microstructure of BC300 is markedly different, consisting of irregularly shaped, loosely aggregated particles with highly rough and porous surfaces. Fine particles and plate-like fragments are dispersed throughout the matrix. The structure is more disordered, and the boundaries between particles are less distinct. Such morphology reflects a lower degree of carbonization and the retention of volatile organic matter and original biomass features. The increased surface roughness and porosity are consistent with an underdeveloped carbon framework, which may negatively affect mechanical performance but could enhance water retention or reactivity depending on the application [60].

In contrast, BC500 displays a more compact and consolidated structure, with angular, faceted particles, smoother surfaces, and sharper edges. These features indicate higher thermal decomposition, partial graphitization, and the collapse or fusion of pores due to the removal of volatiles. The reduced porosity, increased uniformity, and structural densification are typically linked with enhanced mechanical stability and durability in cement-based systems [76].

The comparison clearly demonstrates that increasing the pyrolysis temperature leads to substantial morphological transformations, improving the biochar’s structural compactness and homogeneity, which is critical for its application in cementitious systems.

The elemental composition of BC produced at 300 °C and 500 °C was analyzed using a Rigaku NEX DE X-ray fluorescence (XRF) system combined with a JEOL JCM-7000 scanning electron microscope (SEM). Table 4 presents the elemental composition of BC300 and BC500. It is evident that the sequestered carbon content significantly increases in BC500, accompanied by a low content in oxygen with oxygen-to-carbon ratio (O/C) significantly lower in the case of BC 500 °C, which also indicates higher stability [77].

#### 3.1.7. Potential of Hydrogen (pH) of BC and Electrical Conductivity, µS/cm

BC contains a moderate level of soluble ions. In concrete, high EC might indicate the presence of soluble salts that could contribute to efflorescence or reduce long-term durability. For concrete, the mildly alkaline pH may influence the setting and hydration processes, potentially enhancing the material’s bonding properties with cementitious compounds [78]. Table 5 and Table 6 summarize the pH values of BC300 and BC500, along with the electrical conductivity measurements of the biochar samples. 

The enhanced performance of cement mortars incorporating biochar can be attributed to several synergistic mechanisms.

✓ Firstly, biochar’s porous structure and high-water retention capacity enable it to act as an internal curing agent: it absorbs excess mixing water and gradually releases it during hydration, thereby reducing autogenous shrinkage and promoting more complete formation of hydration products such as calcium silicate hydrate (C–S–H) gel [79,80].
✓ Secondly, the porous morphology and high surface area of milled biochar refine the mortar’s pore structure by filling capillary voids and disrupting pore connectivity, which improves durability and lowers permeability [32].
✓ Thirdly, biochar surfaces, rich in oxygen-containing functional groups, facilitate strong interfacial bonding with the cement matrix and serve as nucleation sites for hydration products, including C–S–H and ettringite: this enhances the interfacial transition zone (ITZ) and improves load transfer across the composite [32].

Milled biochar produced at higher temperatures, such as 500 °C in our study, exhibits higher surface area and more developed microporosity, which magnifies these effects and supports stronger mechanical properties, consistent with the improved compressive strength and bonding observed in previous studies [79].

#### 3.1.8. Sustainability and Practical Implications of Biochar Use in Cementitious Mortars

Although the pyrolysis process used to produce biochar requires energy input, the temperatures employed in this study (300–500 °C) are significantly lower than those in traditional cement clinker production (~1450 °C) [81]. Thus, the energy demand for biochar production is comparatively modest. Incorporating malt-derived biochar as a supplementary additive, not as a cement replacement, can enhance the mechanical performance of cementitious mortars, potentially extending service life and enabling optimized structural designs. These improvements may reduce maintenance frequency and overall material consumption throughout the structure’s lifespan. Additionally, valorizing malt waste into value-added biochar aligns with circular economy principles by promoting resource efficiency and waste reduction. Therefore, the use of biochar in cementitious materials presents a promising approach to sustainable construction practices.

#### 3.1.9. Synergistic Effect of Melflux Superplasticizer and Biochar on Cement Performance

The combined use of Melflux 5581F superplasticizer and biochar demonstrated a notable synergistic effect, significantly enhancing the mechanical performance of cement pastes and mortars.

This effect can be attributed to the complementary mechanisms of the two additives. Melflux reduces water demand and improves particle dispersion through steric hindrance and electrostatic repulsion. This enhances the workability and packing density of the cementitious matrix. Meanwhile, biochar provides nucleation sites due to its high specific surface area and interacts chemically with hydration products through its functional groups [82,83].

The improved dispersion of biochar particles in the presence of Melflux prevents agglomeration and promotes more uniform hydration, resulting in denser microstructure, enhanced calcium–silicate–hydrate (C–S–H) development, and higher compressive strength [79,80].

## 4. Properties of Cement Mortars with BCs

The compositions of the cement mortars mixed with varying concentrations of BC produced at 300 and 500 °C, along with a summary of the results from the 7 and 28-day tests, are presented in Table 7.

### 4.1. Water Absorption of Cementitious Mortar

**Figure 10:**
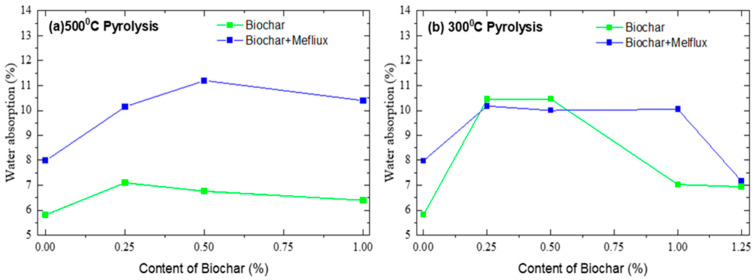
Water absorption of cement mortars made with pyrolyzed BCs at 500 and 300 °C (28 days).

The test samples were stored for 28 days at a temperature of (20 ± 2)°C and a relative humidity of 98%. After this period, the water absorption values were measured for all BC300 and BC500 concentrations (Figure 10) and for the reference samples 1 and 1′.

As a general trend, water absorption increases in the presence of plasticizers and BCs, both for particles produced at 300 and 500 °C, probably due to the discontinuous lattice formed inside the mortars [46]. BCs produced at 300 °C increase the water absorbed in all the samples, with and without the plasticizer; BCs produced at 500 °C reduce the water absorption in samples without plasticizer.

High-water-absorption cementitious materials promote the hydration reaction and significantly increase the durability of the material. According to the results of the specific surface area study of BC, the specific surface area (SSA) of BC500 was 110.402 m^2^/g and exhibited higher hydrophilicity, contributing to the formation of a denser microstructure in the cement mortar. This characteristic may contribute to the reduction in water absorption over time.

### 4.2. Compressive Strength of Cementitious Mortar with BC

**Figure 11:**
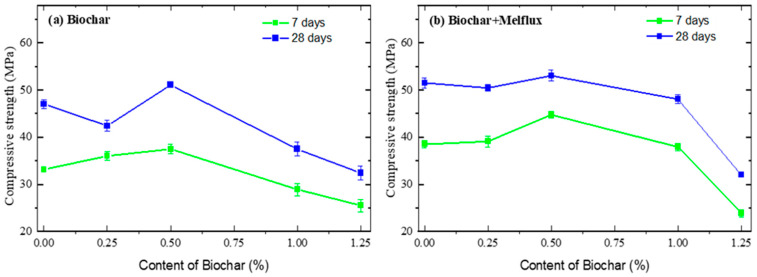
Compressive strengths of cement mortars made with BC pyrolyzed at 300 °C.

**Figure 12:**
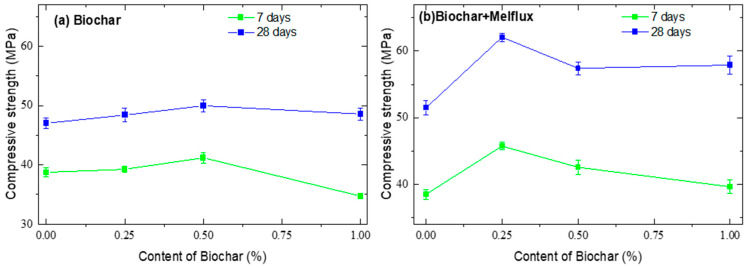
Compressive strengths of cement mortars with BC pyrolyzed at 500 °C.

In Figure 11 and Figure 12, the compressive strengths of cementitious mortars are presented.

Figure 11 shows the compressive strengths of samples prepared with BC pyrolyzed at 300 °C compared to the reference sample (0% BC). Data are reported in Table 7.

The trend indicates an initial increase in the mechanical properties, both after 7 and 28 days, followed by a decrease. This trend is more or less the same for samples prepared with the addition of plasticizer, even though the compressive strength values are higher than without plasticizer.

Figure 12 shows the compressive strength of samples prepared with BC500. After 7 days of curing, the trend is similar to that observed for the samples with BC300. After 28 days, the trend is more homogeneous, and the compressive strength in samples with the addition of BC is generally higher than the values obtained from the samples without BC. Even in this case, the plasticizer seems to increase the mechanical properties.

The trend reported in Figure 11 and Figure 12 is often attributed to a balance between BC’s positive and negative effects on concrete properties. The formation of hydration products, including hydrated calcium aluminosilicate (C-(A)S-H), ettringite (AFt), and calcium hydroxide (CH), necessitates substantial quantities of water to participate in the reaction [76,84,85,86]. BC has a large pore structure that provides superior water absorption and retention capabilities compared to conventional admixtures. Hydrogen bonds allow water to be absorbed and retained in the pore structure. BC has a high capacity to retain water when its pores are between 2.349 nm for BJH and 8.653 nm for D-H in size and pyrolyzed at 5000C. This water is then gradually released during the hydration stage, which promotes the hydration reaction and facilitates internal solidification, leading to a high compressive strength․ That’s why the compressive strengths of BC500 cement mortars were higher compared to BC300, whose pore size ranged from 48.021 to 49.906 nm. From that also emerges a possible complexity in adding BC to concrete, how the ideal amount of BC may differ based on concrete type, application, and other variables [87,88].

BC500 exhibits a finer pore size distribution (2.3–8.6 nm), which allows it to retain and gradually release absorbed water during cement hydration, acting as an internal curing agent. This sustained moisture availability promotes more complete hydration of cement phases, resulting in a denser and stronger matrix. Additionally, BC500 possesses a higher concentration of oxygen-containing functional groups (e.g., hydroxyl, carboxyl) on its surface, enhancing chemical interactions with cement hydration products such as calcium silicate hydrate (C–S–H). These functional groups facilitate nucleation sites for hydration product growth and improve the interfacial transition zone (ITZ) between biochar particles and the cement matrix, leading to better particle packing and reduced porosity. Moreover, the increased specific surface area of BC500 provides more active sites for these reactions, further contributing to the improvement of mechanical strength. Thus, the superior compressive strength of BC500-modified mortars results from a synergistic effect of its pore structure, surface chemistry, and graphitization degree, which collectively optimize hydration kinetics and microstructural development.

The relationship between water absorption and compressive strength in BC-modified mortars can be understood through the dual role of biochar’s pore structure and water retention capacity. Biochar, particularly BC500 with its finer pore size distribution, acts as an internal curing agent by absorbing and gradually releasing water during the cement hydration process. This sustained moisture availability supports more complete hydration of cement phases, resulting in the formation of a denser and more cohesive microstructure. Consequently, the reduction in capillary porosity and refinement of the interfacial transition zone (ITZ) lead to decreased water absorption and enhanced mechanical strength. Conversely, BC with larger pore sizes or higher hydrophobicity (e.g., BC300) may increase water absorption due to less efficient internal curing and more discontinuous pore networks, which can adversely affect strength. Therefore, the interplay between water absorption and compressive strength is largely governed by biochar’s ability to regulate moisture availability and pore connectivity, directly influencing hydration kinetics and microstructural development.

The potential for producing BC from unutilized biomass presents a viable approach to addressing the challenges posed by climate change. The feasibility of incorporating BC into concrete and mortars is a significant consideration when assessing its potential contribution to the construction industry. Although the prospective utilization of BC may contribute to the mitigation of various environmental issues, further evaluation of its economic viability and practical applicability is necessary.

Before considering the use of BC in cementitious composites as a potential advantage in civil engineering, several critical factors must be taken into account:

✓ Biomass as a waste and its potential utilization: The escalation of environmental pollution and the emergence of environmental issues have necessitated the exploration of environmentally benign, renewable, utilizable, and alternative sustainable resources. Significant attention is directed towards industrial waste, particularly waste derived from biomass materials, which can serve as a foundation for biofuel, energy production, and additives in construction materials (concrete, mortar), thereby addressing the increasing demands of humanity. From the biomass material, specifically the malt utilized in this investigation, it is feasible to obtain an additive—BC—through the process of thermochemical degradation in an oxygen-deficient environment. This BC can be incorporated into mortar or concrete mixtures, thereby enhancing the physical and mechanical properties of these composite materials [89,90]. Given that concrete is the primary building material in modern construction, incorporating biomass-derived BC into these mixtures can help utilize accumulated industrial waste and remediate polluted areas [91,92].
✓ Costs and advantages of producing BC: The process of producing BC from biomass is influenced by several factors, including the type of raw materials used, the pyrolysis technology applied, the amount of energy consumed, and the volume of BC generated. Additionally, the potential applications of BC in concrete and construction mortar, as well as the efficiency of the BC production technology compared to other building materials, play significant roles in this process [93,94]. The availability of affordable biomass, specifically the malt used in this work, is crucial. This approach will lower the production costs of BC, making it more practical for widespread use in the construction industry. As a result, it will improve the quality of the final products and significantly reduce negative environmental impacts. By aligning the economic benefits of BC with its environmental advantages, we can explore its potential in construction, paving the way for sustainable building practices [91,95].
✓ Effectiveness of using BC in cement composites: BC is an eco-friendly material that contributes to solving various environmental challenges, such as reducing greenhouse gas emissions and aiding in wastewater treatment. For many years, it has also been used as a soil additive to improve agricultural productivity [96]. While its use in the construction industry is relatively recent, BC holds considerable promise for sustainable development. Its high carbon content, porous structure, and large specific surface area make it highly effective in improving the properties of concrete and mortar compositions. Additionally, incorporating BC into construction materials can help reduce CO_2_ emissions, contributing further to environmental sustainability. In BC -cement composites, BC can control moisture content and promote CO_2_ diffusion during rapid carbonation. According to references [97,98,99], the characteristics of BC cement composites can be improved by combining BC with CO_2_ curing. This innovative method of combining BC and CO_2_ curing offers promise for creating green building materials.
✓ Environmental Implications։ Although a full life cycle assessment (LCA) was not performed in this study, a preliminary estimation of the CO_2_ impact associated with incorporating 1 wt.% barley malt biochar was conducted based on values reported in the literature. Biochar was added as a supplement rather than as a replacement for cement; therefore, direct CO_2_ emission reductions from cement substitution were not considered. Nevertheless, the environmental impact of biochar addition can be approximated by balancing emissions from pyrolysis with the carbon sequestration potential of biochar. Based on life cycle inventories published in the Supplementary Information of [100], slow pyrolysis typically emits 0.6–1.5 kg CO_2_ per kg of biochar, depending on feedstock and system efficiency. Meanwhile, [101] indicates that high-temperature biochars often retain 70–80% of the original carbon in a stable form-equivalent to roughly 2.5–3.0 kg CO_2_ sequestered per kg. Assuming an addition rate of 10 kg of biochar per metric ton of mortar, this corresponds to an estimated net CO_2_ reduction of approximately 10–19 kg per ton.

This preliminary assessment supports the potential environmental benefit of using malt-derived biochar in cementitious materials; however, it also highlights the need for a comprehensive LCA in future studies to validate sustainability claims.

## 5. Conclusions

This study explored the incorporation of biochar (BC) derived from malt through pyrolysis at 300 °C (BC300) and 500 °C (BC500) into cement pastes and mortars at varying replacement levels relative to cement weight. The primary objective was to evaluate the effects on mechanical performance and microstructural properties.

✓ BC300 particles exhibited greater hydrophobicity compared to BC500. BC500 displayed fewer structural defects, higher graphitic ordering, a larger specific surface area, smaller pore diameters, and a significantly lower oxygen-to-carbon (O/C) ratio than BC300.
✓ The incorporation of BC300 at 0.25–1.25 wt.% enhanced compressive strength by up to 13% at 7 days (from 33.16 MPa to 37.48 MPa) and 8.6% at 28 days (from 47.02 MPa to 51.07 MPa) compared to the reference. Similarly, BC500 at 0.25–1 wt.% increased compressive strength by 16.3% at 7 days and 6% at 28 days.
✓ The addition of superplasticizer Melflux 5581F (0.05 wt.% of cement) further enhanced performance. With BC300, compressive strength increased by over 16% at 7 days and 3% at 28 days. BC500 exhibited a strength increase of 18.8% at 7 days and 20.6% at 28 days, reaching 62.09 MPa.
✓ Compared to conventional admixtures, BC demonstrated superior water absorption and retention due to its well-developed pore structure. Pores ranging from 2.349 to 8.653 nm facilitated water retention via hydrogen bonding, enabling gradual water release during cement hydration. This contributed to improved internal curing and enhanced hydration reactions.
✓ BC500’s functional groups (hydroxyl, carboxyl) contributed to improved chemical bonding with hydration products, while its high specific surface area provided nucleation sites for calcium–silicate–hydrate (C–S–H) formation. These microstructural advantages resulted in better packing density, lower water absorption, and superior compressive strength.
✓ Even small dosages (≤1.25 wt.%) of malt-derived biochar can significantly enhance the strength, hydration, and durability of cementitious composites. These results underscore biochar’s potential as a sustainable, low-carbon additive for green construction materials.
✓ Further research should explore the long-term durability, freeze–thaw resistance, acid resistance, and CO_2_ uptake capacity of BC-modified mortars, as well as the performance of biochar derived from various agricultural or food-processing residues.

## Figures and Tables

**Table 1:** Physical, mechanical properties and chemical composition of cement (from commercial datasheet).

Material		Characteristics						
		Standard Consistency, %	Specific Gravity, g/cm^3^		Blaine Fineness, cm^2^/g		Setting Time, Min	Compressive Strength, MPa (28 Days)
Cement 52.5 N		26	3.1		4550		Initial 60 Final 360	52
**Chemical Composition of Cement (wt.%)**								
**SiO_2_**	**CaO**	**Al_2_O_3_**	** Fe_2_O_3_**	**MgO**	**SO_3_**	**Loss on Ignition**	**Insol. Residue**	**Free** **CaO**
20.4	61.4	3.8	4.1	2.4	1.9	2.0	3.1	0.9

**Table 2:** Physical properties of sand (from commercial datasheet).

Material	Sieve Residues, %					Size Modulus, Mk	Bulk Density, kg/m^3^	Real Density, g/cm^3^
	2.5	1.25	0.63	0.315	0.16			
River sand	17.25	31.51	48.84	74.32	93.28	2.65	1650	2.48

**Table 3:** BET analysis data for BC produced through pyrolysis at 300 °C and 500 °C.

Characteristics	Unit	Unmilled BC		Milled BC	
		BC300	BC500	BC300	BC500
Surface Area (BET)	m^2^/g	0.147	1.081	4.140	110.402
Pore V (BJH)	cm^3^/g	0.048	0.011	0.039	0.011
Pore size (BJH)	nm	83.643	83.521	49.906	2.349
Pore size (D-H)	nm	85.715	101.531	48.021	8.653
Estimated pore length (BJH)	µm	1.74	7.88	1.29	0.21
Estimated pore length (D-H)	µm	1.78	9.58	1.24	0.77

**Table 4:** The elemental and oxide composition of milled BC (%) prepared at 300 °C (BC300) and 500 °C (BC500).

Elements	C	O	P	S	Mn	Cu	Zn	CaO	K_2_O	SiO_2_	Al_2_O_3_	Fe_2_O_3_
BC300	57.83	35.92	1.3	0.31	0.006	0.004	0.01	0.4	0.1	5.4	1.4	0.097
BC500	71.52	22.13	2.3	0.12	0.01	0.008	0.02	0.74	0.2	10.7	2.7	0.158

**Table 5:** BC pH.

BC	pH 1 h	pH 2 h	pH 4 h	pH 24 h
BC300	7.01	7.12	7.06	7.11
BC500	7.11	7.10	7.11	7.12

**Table 6:** BC electrical conductivity, µS/cm.

BC	1 h	2 h	4 h	24 h
BC300	316	312	314	311
BC500	380	383	381	385

**Table 7:** Compositions of cement mortars and averaged data of the main physical and mechanical characteristics.

N	Plasticizer %	BC %	Density g/cm^3^	Water Absorption %	Compressive Strength, MPa	
					7 Days	28 Days
**1**	-	-	2.09	5․82	33․16	47․02
**1′**	0․05	-	2.11	7․98	38․49	51․50
	**BC300**					
**2**	-	0.25	2.10	10.18	36.03	42.40
**3**	-	0.50	2.11	10.0	37.48	51.07
**4**	-	1.00	2.14	10.05	28.91	37.49
**5**	-	1.25	2.09	7.17	25.50	31.99
**6**	0.05	0.25	2.13	10.45	39.09	50.46
**7**	0.05	0.50	2.13	10.46	44.75	53.07
**8**	0.05	1.00	2.11	7.03	37.92	48.06
**9**	0.05	1.25	2.13	6.94	23.88	32.39
	**BC500**					
**10**	-	0.25	2.16	7.10	38.56	48.40
**11**	-	0.50	2.17	6.76	36.96	49.96
**12**	-	1.00	2.16	6.40	36.70	48.58
**13**	0.05	0.25	2.08	10.15	45.74	62.09
**14**	0.05	0.50	2.11	11.20	42.57	57.43
**15**	0.05	1.00	2.09	10.40	39.64	57.90

## Data Availability

The original contributions presented in this study are included in the article. Further inquiries can be directed to the corresponding author.
